# Bioelectrical Impedance Changes of the Trunk are Opposite the Limbs Following Acute Hydration Change

**DOI:** 10.2478/joeb-2022-0005

**Published:** 2022-06-25

**Authors:** Dale R. Wagner

**Affiliations:** 1 Kinesiology and Health Science Department, Utah State University, Logan, UT, USA

**Keywords:** bioimpedance, body composition, body water, dehydration, hyperhydration

## Abstract

This study aimed to evaluate the changes in impedance and estimates of body composition variables obtained from segmental multi-frequency bioelectrical impedance analysis (SMFBIA) following acute hydration change. All participants (N = 11 active adults) had SMFBIA measurements at baseline (euhydration), post-dehydration, and post-hyperhydration in an experimental repeated-measures design. Dehydration and hyperhydration trials were randomized with the opposite treatment given 24 h later. Dehydration was achieved via a heat chamber of 40 °C and 60% relative humidity. Hyperhydration was achieved by drinking lightly-salted water (30 mmol·L^-1^ NaCl; 1.76 g NaCl·L^-1^) within 30 min. Post-measurements were taken 30 min after each treatment. Despite changes in mass post-dehydration (Δ = -2.0%, p < 0.001) and post-hyperhydration (Δ = 1.2%, p < 0.001), SMFBIA estimates of total body water (TBW) did not change significantly across trials (p = 0.507), leading to significant differences (p < 0.001) in SMFBIA-estimates of body fat percentage across trials. Dehydration resulted in a significant (p < 0.001) 8% decrease in limb impedances at both 20 kHz and 100 kHz. Hyperhydration increased limb impedances only slightly (1.5%, p > 0.05). Impedance changes in the trunk followed an opposite pattern of the limbs. SMFBIA failed to track acute changes in TBW. Divergent impedance changes suggest the trunk is influenced by fluid volume, but the limbs are influenced by ion concentration.

## Introduction

The use of bioelectrical impedance analysis (BIA) to estimate human body composition parameters such as total body water (TBW), fat-free mass (FFM), and body fat percentage (%BF) dates back to the mid-1980s [1, 2, 3, 4]. The principles, assumptions, and clinical applications of the BIA method were thoroughly reviewed by Kushner [5]. These early studies used a single-frequency (50 kHz) BIA device with gel electrodes placed on the hands and feet in a tetrapolar configuration with the study participant lying in a supine position. Soon after, researchers began experimenting with segmental BIA, evaluating the limbs independently of the trunk [6], and multi-frequency BIA to distinguish between intracellular and extracellular water [7]. By the end of the 20^th^ century, user-friendly hand-to-hand (hand-held) [8] and foot-to-foot (scale) [9] BIA devices with plate or contact electrodes were available for home use. Today, these BIA variations have merged such that user-friendly segmental multi-frequency BIA (SMFBIA) devices are commonplace in research laboratories, clinical settings, and fitness centers.

SMFBIA was validated against isotope dilution for TBW measurement [10,11], and against DXA [12,13] and a 4-compartment model [10] for FFM. The SMFBIA method is quick, easy to administer, non-invasive, and less expensive than many other body composition methods. For these reasons, SMFBIA is an appealing assessment method. Despite these advantages, numerous factors can affect the validity of this method. For example, accurate body positioning and electrode placement are critical for valid measurements [14,15]. Another variable known to affect the impedance of electrical current, and consequently the body composition measures derived from SMFBIA, is hydration status [16].

A substantial volume of research exists on the effect hydration status and hydration change has on bioimpedance measures; however, some questions remain. For example, O’Brien et al. [16] reviewed a number of hydration change studies that used single-frequency BIA and SMFBIA, and they found equivocal results with resistance to the electrical current both increasing and decreasing in response to acute hydration change. More importantly, all of the previous SMFBIA research on this topic has been limited to total body results; segmental impedance values following acute hydration change have not been reported. This segmental data could help elucidate the confounding question of what happens to impedance values following an acute change in hydration status. Thus, the purpose of this study was to evaluate the change in SMFBIA-estimated body composition variables (TBW, FFM, and %BF) as well as segmental impedance values following both acute dehydration and acute hyperhydration.

## Materials and methods

The SMFBIA data collection was a sub-investigation of the acute effects of hyperhydration and dehydration on A-mode ultrasound measures of subcutaneous fat thickness [17]; thus, the methods presented here are similar to those presented previously [17] with the exception that SMFBIA information is included, and ultrasound information is excluded.

### Sample

The study was open to adults, aged 18-65 y, and recruitment was via word-of-mouth within a university exercise science department and an advertisement posted at the university’s fitness center. Exclusion criteria included pregnancy, a self-reported inability to be confined in a hot environment or to drink a large volume of lightly-salted water, current use of an NSAID, or prior history of hyponatremia.

### Procedures

Participants came to the laboratory on two consecutive days. They followed a pre-session euhydration protocol of drinking 10 ml∙kg^-1^ water approximately 2 h before arriving at the lab, maintained their typical daily routine, and refrained from exhaustive exercise prior to each trial. Upon arrival at the lab, participants emptied their bladder and provided a urine sample. Urine specific gravity (USG) was measured with a refractometer (Uricon-N, Atago, Tokyo, Japan) to verify euhydration status. A USG of < 1.020 was the criterion for euhydration [18]. Height was measured to the nearest 0.1 cm with a custom-made, wall-mounted stadiometer. Body mass was measured to the nearest 0.02 kg with a digital scale (DI/10, Wedderburn Scales Ltd., Dunedin, New Zealand). Male participants wore shorts only, while female participants wore shorts and a sports bra for all measurements.

SMFBIA measures were taken with an InBody 230 analyzer (Seoul, South Korea) following the manufacturer’s instructions for body position [19]. This included standing erect with the feet positioned on the foot electrodes and the arms slightly abducted with the hands grasping the electrodes on the handrails. This SMFBIA model provided impedance for five segments (right and left arms, right and left legs, and trunk) at two frequencies (20 kHz and 100 kHz). TBW, FFM, and %BF were estimated from the manufacturer’s proprietary formulas.

Participants were randomly assigned to dehydration or hyperhydration, with the opposite treatment applied during the subsequent trial. Dehydration took place in an environmental chamber with the temperature and relative humidity held constant at 40 °C and 60%, respectively. Participants had access to a cycle ergometer and treadmill, and exercise was ad libitum. A scale was available for participants to self-monitor changes in mass. Participants remained in the chamber until approximately 2% of body mass was lost or 2 h elapsed, whichever occurred first. Post-dehydration measurements occurred 30 min after exiting the chamber.

The hyperhydration protocol required participants to drink 2% body mass of lightly-salted water (30 mmol∙L^-1^ NaCl; 1.76 g NaCl∙L^-1^). They were encouraged to finish this volume within 30 min. Once consumed, they waited an additional 30 min before beginning the post-treatment measurements.

Participants emptied their bladder, and body mass was measured. The change in mass (Δmass) between the baseline and this post-treatment measurement confirmed and quantified dehydration and hyperhydration. SMFBIA measurements were repeated following the same standards described for baseline testing.

### Statistical analyses

Data were checked for normality by visual inspection of plots and the Shapiro-Wilk test. Mean differences in body mass, TBW, FFM, %BF, and impedance for each body segment at both 20 kHz and 100 kHz between the two baseline measurements and the dehydration and hyperhydration post-treatments were evaluated using repeated-measures ANOVA. If the assumption of sphericity was violated, the Greenhouse-Geisser correction was used. When the F-score was significant, pairwise comparisons were made using the least significant difference. Statistical significance was accepted as *p* < 0.05. Effect sizes of the pairwise differences were reported as Cohen’s *d* with the benchmarks of 0.2, 0.5, and 0.8 defining small, medium, and large effects, respectively [20]. All statistical analyses were performed with SPSS version 25 (IBM, Armonk, NY).

### Informed consent

Informed consent has been obtained from all individuals included in this study.

### Ethical approval

The research related to human use has been complied with all relevant national regulations, institutional policies and in accordance with the tenets of the Helsinki Declaration, and has been approved by the authors’ institutional review board or equivalent committee.

## Results

Eleven recreationally-active adults (8 male, 3 female), ranging in age (19 to 54 y; 27.1 ± 10.5 y), height (156.5 to 188.8 cm; 176.2 ± 11.5 cm), mass (49.2 to 120.3 kg; 78.0 ± 20.6 kg), and body mass index (20.3 to 33.5 kg∙m^-2^; 24.7 ± 3.9 kg∙m^-2^) completed the study. Measured data from the dehydration trial are in Table 1, and data from the hyperhydration trial are in Table 2. All participants began each trial with USG < 1.020. The difference in USG was not significant between trials (Δ = 0.0035, 95%CI = -0.0074 to 0.0005, *p* = 0.081), suggesting similar euhydration at the start of each session. Body mass was similar at the start of each trial (Δ = 0.03 kg, 95%CI = -0.60 to 0.66 kg, *p* = 1.000) but decreased significantly post-dehydration (Δ = -2.0% or -1.56 kg, 95%CI = -2.13 to -0.99 kg, *p* < 0.001) and increased significantly post-hyperhydration (Δ = 1.2% or 0.90 kg, 95%CI = 0.68 to 1.11 kg, *p* < 0.001). Six of the 11 subjects stayed in the heat chamber for the full 2 h, while the others achieved the desired mass loss in less time. The average time to dehydrate 2% was 99 ± 28 min. The average amount of water consumed during the hyperhydration trial was 1,560 ± 413 ml.

**Table 1:** Mean ± SD of the sample (N = 11) for the dehydration trial.

Variable	Baseline	Post-Dehydration	Change from baseline (%)	Pairwise p-value	Cohen's *d* effect size
Body mass (kg)	78.0 ± 20.4	765 ± 20.2	-2.0±0.6	<0.001	2,71
TBW (kg)	48.9 ± 12.8	49.2 ± 12.5	0.9 ± 2.5	0.374	0.28
FFM (kg)	66.9 ± 17.7	67.1 ± 17.1	0.6 ± 2.5	0.617	0.16
Body fat (%)	14.1 ± 7.3	11.6 ± 8.1	-24.6 ±23.6	0.004	1.11
Z RA 20 kHz (Ω)	306.2 ± 60.4	281.4 ± 48.4	-7.7 ± 4.6	0.001	1.37
Z LA 20 kHz (Ω)	309.5 ±62.6	282.9 ± 50.1	-8.2 ± 4.3	<0.001	1.54
Z RL 20 kHz (Ω)	251.1 ± 33.6	232.1 ± 31.0	-7.5 ± 4.2	<0.001	1.82
Z LL 20 kHz (Ω)	250.6 ±33.8	230.4 ±29.1	-7.9 ± 4.4	< 0.001	1.70
ZTR 20 kHz (Ω)	21.1 ± 3.0	22.5 ± 3.1	6.9 ± 8.8	0.029	0.77
Z RA 100 kHz (Ω)	270.3 ± 57.3	246.8 ±46.1	-8.2 ± 4.9	0.001	1.37
Z LA 10D kHz (Ω)	273.8 ± 59.7	249.4 ± 47.5	-8.4 ± 4.9	0.001	1.42
Z RL 100 kHz(Ω)	219.0 ±31.7	202.7 ±28.8	-7.3 ± 4.3	<0.001	1.75
Z LL 100 kHz (Ω)	218.1 ± 31.7	200.9 ± 27.5	-7.7 ± 4.3	<0.001	1,71
Z TR 100 kHz (Ω)	17.1 ± 2.9	18.2 ± 3.1	6.6 ± 8.1	0.023	0.81

TBW = total body water; FFM = fat-free mass; Z = impedance; RA = right arm; LA = left arm; RL = right leg; LL = left leg; TR = trunk

**Table 2:** Mean ± SD of the sample (N = 11) for the hyperhydration trial.

Variable	Baseline	Post-Hyperhydration	Change from baseline (%)	Pairwise p-value	Cohen's *d* effect size
Body mass (kg)	78.0 ± 20.6	78.9 ± 20.8	1.2 ± 0.2	< 0.001	4.18
TBW (kg)	48,8 ± 12.7	49.0 ± 13.0	0.3 ± 09	0.240	0.38
FFM (kg)	66.7 ± 17.4	67.0 ± 17.9	0.3 ± 0.9	0.242	0.38
Body fat (%)	13.8 ± 8.4	14.7 ± 8.1	10.8 ± 15.6	0.024	0.80
Z RA 20 kHz (Ω)	309.6 ± 58.3	314.9 ± 64.7	1.4 ± 2.7	0.079	0.59
Z LA 20 kHz (Ω)	314,0 ± 60.1	318.3 ± 65.2	1.2 ± 2.7	0.149	0.47
Z RL 20 kHz (Ω)	252.8 ± 35.1	257.4 ± 43.3	1.5 ± 3.8	0.168	0.45
Z LL 20 kHz (Ω)	251.4 ± 35.5	255.8 ± 44.0	1.4 ± 4.0	0.195	0.42
Z TR 20kHz(Ω)	21.2 ± 3.3	20.9 ± 3.1	-1.3 ± 2.7	0.099	0.55
Z RA 100 kHz (Ω)	272.2 ± 55.6	276.5 ± 60.8	1.3 ± 2.8	0.100	0.55
Z LA 100 kHz (Ω)	276.8 ± 57.3	280.3 ± 61.5	1.0 ± 2.7	0.179	0.44
Z RL 100 kHz (Ω)	220.5 ± 32.8	224.5 ± 39.2	1.5 ± 3.6	0.141	0.48
Z LL 100 kHz (Ω)	218.5 ± 32.9	222.5 ± 39.5	1.5 ± 3.5	0.131	0.50
Z TR 100 kHz (Ω)	16.9 ± 3.1	16.7 ± 3.1	-1.5 ± 3.2	0.154	0.47

TBW = total body water; FFM = fat-free mass; Z = Impedance; RA = right arm; LA = left arm; RL = right leg; LL = left leg; TR = trunk

**Fig.1:**
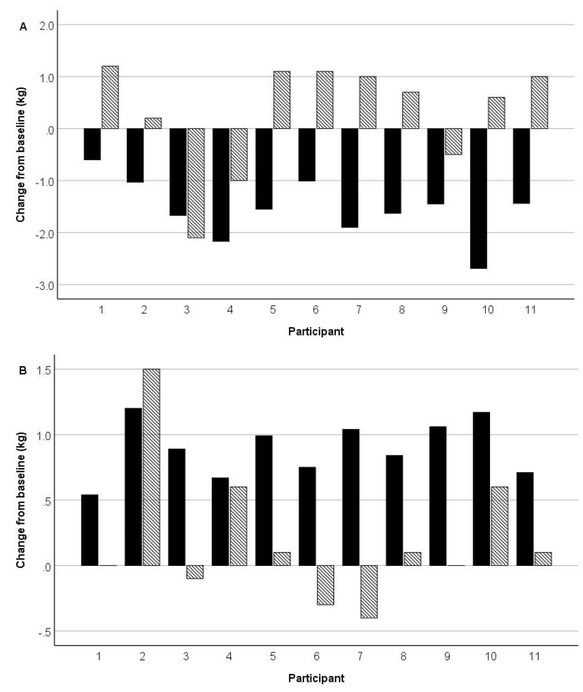
Changes from baseline in body mass (solid bars) and SMFBIA-estimated total body water (hashed bars) following A) dehydration and B) hyperhydration.

SMFBIA estimates of TBW did not change significantly across trials (*p* = 0.507) despite changes of approximately ±2% of body mass following dehydration and hyper-hydration. Correspondingly, estimates of FFM did not change significantly across trials (*p* = 0.796). SMFBIA failed to detect any loss in TBW in 8 of the 11 subjects following the dehydration protocol; in fact, the TBW estimate increased in these subjects (Figure 1a). Similarly, SMFBIA TBW estimate failed to increase more than 0.1 kg in 8 of the 11 subjects following the hyperhydration trial (Figure 1b). Consequently, the estimates of %BF from SMFBIA differed significantly across the 4 measurements (*p* < 0.001). The %BF post-dehydration was significantly lower than the other measurements, which did not differ significantly among each other.

Consistent with the laws of electrical conduction for a capacitive impedance system (negative phase angle) [5], the higher frequency (100 kHz) resulted in less impedance than the lower frequency (20 kHz) for a given body segment, and the arms, with their smaller cross-sectional area, created the most impedance followed by the legs and trunk (Tables 1 and 2). Baseline impedance values were consistent from day-today (*p* > 0.05). Dehydration resulted in a significant decrease (*p* < 0.01) of about 8% in limb impedance at both 20 kHz and 100 kHz.

Although there was a tendency for impedance to increase in the limbs following hyperhydration, this did not reach statistical significance (*p* > 0.05). Impedance changes in the trunk followed an opposite pattern of the limbs with a significant increase from dehydration (*p* < 0.05) and a nonsignificant (*p* > 0.05) trend to decrease following hyperhydration.

## Discussion

The primary finding from this investigation was that the change in TBW estimated from SMFBIA did not match, or even approach, the change in body mass following dehydration or hyperhydration. This finding was expected as others reported that BIA and SMFBIA were not accurate at tracking acute hydration changes [21, 22, 23, 24, 25]. Further, changes in impedance were more pronounced following dehydration than hyperhydration in the present study. One possible reason for this was the greater change in mass following the dehydration trial compared to the hyperhydration trial. Participants changed by approximately 2% of their mass in both trials; however, there was a 30-minute equilibration period following hyperhydration after which participants urinated, thereby reducing the total mass change.

Theoretically, impedance should increase when one is in a state of hypohydration relative to euhydration because there is less fluid to conduct the current. The opposite is true of hyperhydration. However, a review of the literature indicates that the impedance change is often in the direction opposite of expectations following acute dehydration or hyperhydration [16]. This is because the water-electrolyte balance typically is not maintained following acute hydration change, and the electrolyte concentration might have a greater effect on BIA than the change in TBW [16]. Further, physiological changes that typically coincide with exercise-induced dehydration, such as sweating, increased skin temperature, and blood flow redistribution, may transiently alter impedance independent of TBW changes [16].

The most unexpected and novel finding from this study was truncal impedance changes were similar in magnitude (percent change) yet opposite in direction from limb impedance changes in response to hydration changes. Following dehydration, truncal impedance increased, matching the theoretical expectation that a decrease in fluid increases resistance to electrical current flow. However, limb impedance, at both low and high frequencies, consistently and significantly decreased in response to dehydration, suggestive of influence by a high electrolyte concentration. Although the magnitude of change was not great (and not statistically significant) following the hyperhydration trial, truncal impedance decreased consistent with expected fluid change while limb impedance increased consistent with electrolyte change. Thus, from this observation, it appears that fluid or TBW changes influence the trunk while ion concentration influences the limbs following acute hydration change.

The author can only speculate on the reason for the divergence of impedance change between the trunk and limbs in response to acute hydration change. First, it is important to recognize that acute TBW changes are not consistent throughout the body [26,27]. For example, heat exposure shifts water to the extracellular component to facilitate sweating [21,28]. Second, sweat gland density varies widely from one area of the body to another [29], and not all sweat glands are recruited simultaneously [30]. Third, heat and exercise increase skin temperature, which consistently decreases impedance [15,16,21,31]. Fourth, it might take more than 30 min for skin temperature to return to neutral following heat exposure [32]. Taken together, these factors may help explain why truncal and limb impedance values move in opposite directions following acute hydration change. Post-dehydration measurements were made at 30 min of exiting the environmental chamber, and skin temperature at the SMFBIA contact points (hands and feet) could have still been elevated following the heat/dehydration trial, leading to decreased limb impedance. Conversely, following the hyperhydration trial, a majority of the water would have emptied from the gut within 30 min [33], but much of it would have still been in the intestines, thereby increasing the fluid volume of the trunk and decreasing truncal impedance.

Results from the present study suggest that hyperhydration may have less of an influence on SMFBIA impedance values than dehydration. The magnitude of impedance change post-dehydration was statistically significant and greater than post-hyperhydration (not statistically significant). Similarly, other investigators found that excess hydration had either no significant influence on impedance or increased impedance only slightly from euhydration [14,23,34]. Fluid volume is likely a critical factor for inducing an impedance change [23,24]. Fluid intake of ≤ 1,000 mL appears to have negligible impact on impedance [14,34]. Others reported statistically significant changes in BIA-measured body composition variables following acute hydration of 500-750 mL, but they determined that these changes were not clinically meaningful [35,36]. The fluid intake in the present study was substantial, averaging > 1,500 mL; nevertheless, the impedance change remained negligible at ≤ 1.5% at all body segments.

This study has limitations and strengths. Additional measures, such as plasma osmolality and skin temperature, potentially could have helped explain the mechanism underlying why the trunk and limbs responded differently to changes in hydration status. Also, the post-treatment SMFBIA measurement was taken at a single time point (approximately 30 min after either dehydration or hyperhydration). Tracking impedance change over time (e.g., immediate post-treatment, 30 min, 60 min, 90 min, and 120 min) could have aided in determining the optimal time to conduct SMFBIA measures following a rapid alteration in hydration status. Despite these limitations, this is the first paper to identify a contrary impedance response between the limbs and trunk following acute hydration change. Another strength of the study was the inclusion of both a dehydration and hyperhydration trial, as previous investigators of BIA hydration status studies evaluated one or the other, or rehydrated the participants immediately after dehydration. Further, the methods to induce hyperhydration (e.g., fluid volume relative to mass) and dehydration (e.g., temperature- and humidity-controlled chamber) were tightly controlled.

## Conclusion

In conclusion, TBW estimates from SMFBIA failed to match the body mass changes that accompanied acute dehydration and hyperhydration. Further, impedance values at the limbs moved in opposite direction to impedance values at the trunk at both 20 kHz and 100 kHz following acute dehydration. Impedance at the trunk appears to be influenced by fluid volume, while impedance at the limbs appears to be influenced by ion concentration; however, this is speculation, and more research is needed to determine the mechanistic reason for the divergence in SMFBIA impedance values between the trunk and limbs following acute hydration change.
